# Response to gefitinib and erlotinib in Non-small cell lung cancer: a retrospective study

**DOI:** 10.1186/1471-2407-9-333

**Published:** 2009-09-18

**Authors:** Ivette F Emery, Chiara Battelli, Paul L Auclair, Kathleen Carrier, Daniel M Hayes

**Affiliations:** 1 Department of Translational Research, Maine Center for Cancer Medicine, Scarborough, Maine, USA; 2 Department of Internal Medicine, Maine Medical Center, Portland, Maine, USA; 3 Department of Pathology, Maine Medical Center, Portland, Maine, USA; 4 Histology Core, Maine Medical Center Research Institute, Scarborough, Maine, USA

## Abstract

**Background:**

In Non-small cell lung cancer (NSCLC), an overactive epidermal growth factor receptor (EGFR) pathway is a component of the malignant phenotype. Two tyrosine kinase inhibitors (TKIs) of EGFR, gefinitib and erlotinib, have been used with variable benefit.

**Methods:**

We have analyzed outcome data of a population of NSCLC patients that received these TKIs to determine the benefit derived and to define the clinical and molecular parameters that correlate with response. Tumor tissue from a subgroup of these patients was analyzed by immunohistochemistry to measure the expression level of EGFR and four activated (phosphorylated) members of the pathway, pEGFR, pERK, pAKT, and pSTAT3.

**Results:**

Erlotinib was slightly superior to gefitinib in all measures of response, although the differences were not statistically significant. The most robust clinical predictors of time to progression (TTP) were best response and rash (p < 0.0001). A higher level of pEGFR was associated with longer TTP, while the total EGFR level was not associated with response. Higher levels of pAKT and pSTAT3 were also associated with longer TTP. In contrast, a higher level of pERK1/2 was associated with shorter TTP.

**Conclusion:**

These observations suggest the hypothesis that tumor cells that have activated EGFR pathways, presumably being utilized for survival, are clinically relevant targets for pathway inhibition. An accurate molecular predictive model of TKI response should include activated members of the EGFR pathway. TKIs may be best reserved for tumors expressing pEGFR and pAKT or pSTAT, and little pERK. In the absence of molecular predictors of response, the appearance of a rash and a positive first scan are good clinical indicators of response.

## Background

Current chemotherapy combinations for advanced non-small cell lung cancer (NSCLC) have reached a plateau in overall response rate and are never curative. Response rates for first line and second line treatments are 35% and 8%, respectively, and time to progression averages four to six months [1-3]. New therapeutic agents and strategies to maximize the efficacy of current treatments are clearly needed.

In addition to classic cytotoxic agents, approved systemic therapies to treat NSCLC include inhibitors of the epidermal growth factor receptor (EGFR) pathway. The EGFR pathway is a principal transducer of growth and survival signals in lung cancer cells and therefore a logical target for therapy. Gefitinib and erlotinib are reversible inhibitors of the kinase domain of EGFR that compete with ATP for binding to the catalytic pocket. These small molecules inhibit EGFR autophosphorylation and, thus, they inhibit receptor dimerization, and the downstream signaling that would have otherwise stimulated proliferation (through the activation of Erk) and anti-apoptotic mechanisms (through activation of Akt and Stat).

TKIs were developed after accumulating evidence indicated that a large proportion of NSCLC over-expresses the EGF receptor and is likely to be dependent on this pathway for the malignant phenotype [4]. However, TKIs in NSCLC therapy have not produced the gains in survival and time to progression that were anticipated when used alone or in combination with traditional cytotoxic agents [2,3,5]. Given this limitation, one way to increase the effectiveness of these agents is to rationally select patients based on tumor markers that predict response.

The identification of molecular predictors of response to anti-EGFR agents has been difficult. Unlike trastuzumab, whose clinical activity can be predicted by the evidence of over-expression of its target, the ErbB2 receptor, either by fluorescence in situ hybridization (FISH) or immunohistochemistry (IHC) [6], single markers have not produced accurate predictive models of response for anti-EGFR agents such as gefitinib and erlotinib. Early results on the relationship between EGFR protein overexpression or *egfr *gene amplification and response were contradictory and recent large studies indicate that there is no correlation between high EGFR expression by IHC or amplification by FISH and better response to TKIs [5,7-9]. Specific mutations in the *egfr *gene have correlated with the dramatic responses that are sometimes seen in a small percentage of patients treated with these agents. However, these results are confounded by the prognostic value of the mutations, since it has been noted that these patients have a better prognosis, regardless of treatment used, than patients whose tumors do not bear *egfr *mutations [10]. Moreover, the mutations do not identify the large number of patients that achieve more modest responses.

We evaluated data from our lung cancer patients to compare the benefits derived from erlotinib and gefitinib and to identify the clinical parameters that correlated with response. We subsequently analyzed tumor tissue from a subgroup of these patients for a pilot study to evaluate the utility of a combination of markers, downstream from the inactivated EGFR receptor, to predict response to TKIs and correlate outcomes. We based our design on the hypothesis that an accurate predictive model may need several markers, and that markers closer to the malignant phenotype (activated, phosphorylated proteins) may be better predictors than DNA/RNA markers or inactive (unphosphorylated) protein markers. In order to determine which tumor will be susceptible to anti-EGFR therapy, one may need to assess the level of phosphorylated EGFR as well as the level of phosphorylated downstream mediators, Akt, Erk1/2 and Stat3, the cytosolic end points to the three branches of the EGFR signaling pathway. Moreover, clinical data have identified these markers as relevant to the natural history of lung cancer [11-15].

## Methods

### Patient Data

At the Maine Center for Cancer Medicine, clinical data were compiled for 160 NSCLC patients that had received either gefitinib or erlotinib between 1996 and 2002 and organized into a database constructed in FileMaker Pro 8. Recorded variables included age, sex, smoking history, ECOG performance status score, date of diagnosis, stage at diagnosis, treatments, and toxicities. Outcome data included best response, time to progression, survival since start of TKI treatment, and overall survival. Best response to the new therapeutic agent was documented in the first computed tomography (CT) scans done 3-4 months after the start of TKI therapy. Time to progression was defined as the time interval from the initiation of TKI therapy to the time of disease progression as evidenced by CT scan or by a decline in performance status. Survival since TKI treatment was defined as the time interval from initiation of TKI therapy to death.

Paraffin-embedded tumor samples were obtained from a subgroup of patients (n = 32) that had received gefitinib or erlotinib and had adequate histological material. Twenty three of these were primary lung tumor resection samples, six were lymph node samples, and three were samples from other biopsy sites. All samples had been fixed in 10% buffered formalin. Clinical data were kept unavailable during the laboratory analysis until all data were evaluated. This study was approved by the Maine Medical Center Institutional Review Board.

### Immunohistochemistry

Formalin-fixed, paraffin-embedded tumor samples were cut in 4 μm-thick sections. Consecutive sections were then treated with primary antibodies purchased from Cell Signaling Technologies (Beverly, MA, USA) to visualize phospho-EGFR (Tyr1173) (53A5 rabbit mAb), phospho-Akt (Ser473) (736E11 rabbit mAb), phospho-Stat3 (Tyr705) (D3A7 rabbit mAb) and phospho-Erk1/2 (Thr202/Tyr204) (20G11 rabbit mAb), according to the manufacturer's instructions. Primary antibodies were visualized with biotinylated secondary antibodies, streptavidin/HRP enzyme complex and DAB chromogen. The slides were counterstained with hematoxylin. Total EGFR levels were obtained from sections sent to NorDx Laboratories and stained on a Ventana BenchMark XT automated stainer using anti-EGFR mouse monoclonal antibody 3C6 and Basic DAB Detection kit (Ventana Medical Systems, Tucson, AZ, USA). Protocols were pre-tested using positive and negative control cell pellets for each anti-phospho protein antibody, available through Cell Signaling Technologies. Sections were scanned at low magnification and portions containing tumor tissue were delineated by the study pathologist (PA). Specimens were evaluated by light microscopy by two independent observers and scored based on a semiquantitative approach of percentage of positive tumor cells (0-100%), multiplied by staining intensity (0 = negative, 1 = weak, 2 = moderate, 3 = strong). In cases in which there were tumor cell populations within one sample with clear distinction in staining intensities, each region's intensity was multiplied by the appropriate percentage and added to produce the sample score. The final percentage of cells staining and score for each slide was calculated as the average between the percentage and scores of the two observers. For example, a sample that was scored by one observer as having 100% of cells staining, with 50% of them staining at intensity 1 and 50% at intensity 2, and was scored by the second observer as having 80% of cells staining, with 70% of them staining at intensity 1 and 10% at intensity 2, would receive a score of 150 [(50 × 1) + (50 × 2)] from observer one, and a score of 90 [(70 × 1) + (10 × 2)] from observer two. Accordingly, in this sample, the final percentage of cells staining would be 90% (mean between 100% and 80%), and the final score would be 120 (mean between 150 and 90). In this manner, a total score range of 0-300 was generated for each sample, where 0 was classified as no expression, 1-10 was classified as low expression, 11-100 was classified as moderate and 101-200 was classified as moderate-high expression level, and scores over 200 were classified as high expression level. The distribution of staining (membranous, cytoplasmic, or nuclear) was recorded. Positive expression of pERK1/2 in stromal fibroblasts and endothelial cells of tumor tissue was used as an internal standard of tissue quality and antigen preservation.

### Statistical Analysis

Univariate analysis between time to progression and survival according to best response, and appearance of rash were conducted using the Kaplan-Meier method. For the patients with tissue sample (n = 32), the associations between time to progression and survival according to level of expression of four phospho-proteins, and total EGFR were investigated using Rapid Insight Analytics statistics program software, and box plots analyses with Excel and XLSTAT program software.

## Results

### Response to tyrosine kinase inhibitors

Between 1996 and 2002, 160 patients with NSCLC received a tyrosine kinase inhibitor during treatment at the Maine Center for Cancer Medicine. One hundred and fifteen (115) patients received gefinitib and forty five (45) received erlotinib. We analyzed the baseline characteristics of these two populations and found them to be nearly equivalent in regards to age, smoking history, ECOG performance, stage, and number of previous line of chemotherapy. The population of erlotinib users had a higher proportion of females as compared to the population of gefitinib users (Table 1).

**Table 1:** Patient characteristics

	Gefitinib		Erlotinib	
	**No**	**%**	**No**	**%**

TOTAL	115		45	

**Gender**				

*Female*	55	**48**	30	**67**

*Male*	60	**52**	15	**33**

**Age**				

*Avg. age*	66.53		66.76	

*Range*	45-86		35-82	

**Smoking history**				

*Smokers*	109	**95**	39	**87**

*Non-smokers*	5	**1**	4	**9**

*Unknown*	1	**1**	2	**4**

*Avg. pack-years*	43.6		42.2	

**Histology**				

*Adenocarcinoma*	54	**47**	15	**33**

*Squamous*	27	**23**	9	**20**

*Bronchioalveolar*	8	**6**	2	**4**

*Large cell*	6	**5**	0	**0**

*Mixed*	2	**2**	0	**0**

*NSC not further specified*	17	**15**	20	**44**

**Avg. ECOG**	1.37		1.37	

**Avg. previous lines of chemo**	1.26		1.13	

As assessed by CT scans within 3-4 months after the initiation of therapy or by clinical examination when declining performance status prevented scanning, most patients in the gefitinib group had progressive disease (65%), but a quarter had stable disease (26%) and a small minority had a response (3% CR and 3% PR). In contrast almost half the patients in the erlotinib group experienced stable disease (47%) and 10% had a response (5% CR and 5% PR), while 41% showed progressive disease. The longest time to progression was 35 months for erlotinib and 72 months for gefitinib. The patient that experienced no progression for over 6 years was a Caucasian female with a 100 pk/year smoking history.

**Figure 1:**
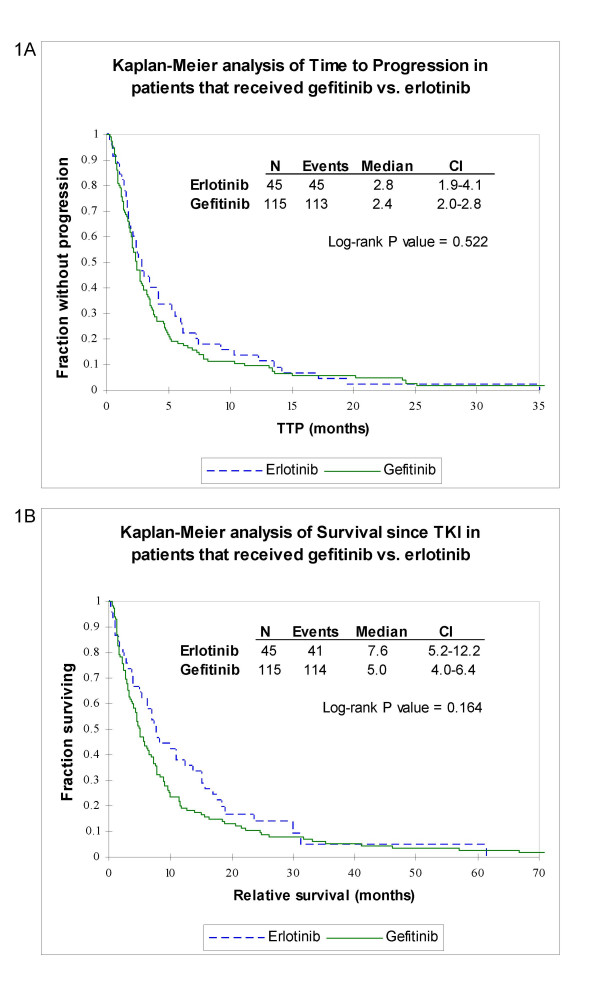
**Gefinitib vs. erlotinib**. Kaplan-Meier analyses of time to progression (a) and survival since initiating TKI therapy (b) in months among gefitinib users (solid line) and erlotinib users (dashed line).

The most common side-effects for both groups were rash, followed by diarrhea, nausea/vomiting and fatigue (Table 2). In the gefitinib group, four patients had significant anorexia and three had clinically evident interstitial lung disease, with swelling/scarring of alveoli and interstitium. Twenty percent of patients in the gefitinib and 17% in the erlotinib group discontinued therapy due to the side-effects. Median time to progression and survival since TKI treatment were, respectively, 2.8 and 7.6 months in the erlotinib group, and 2.4 and 5.0 months in the gefitinib group, but these differences were not statistically significant (Figures 1a-b).

**Table 2:** Side effects

	Gefitinib				Erlotinib			
	**Grade 1-2**		**Grade 3-4**		**Grade 1-2**		**Grade 3-4**	

	**No**	**%**	**No**	**%**	**No**	**%**	**No**	**%**

*Skin rash*	40	**35**	6	**5**	24	**53**	2	**4**
*Diarrhea*	35	**30**	8	**7**	15	**33**	2	**4**
*Nausea/Vomiting*	8	**7**			7	**16**	1	**2**
*Fatigue*	5	**4**	2	**2**	3	**7**	2	**4**
*Anorexia*	4	**3**	1	**1**				
*Instertitial Lung Disease*	3	**3**						

### Clinical predictors of response

**Figure 2:**
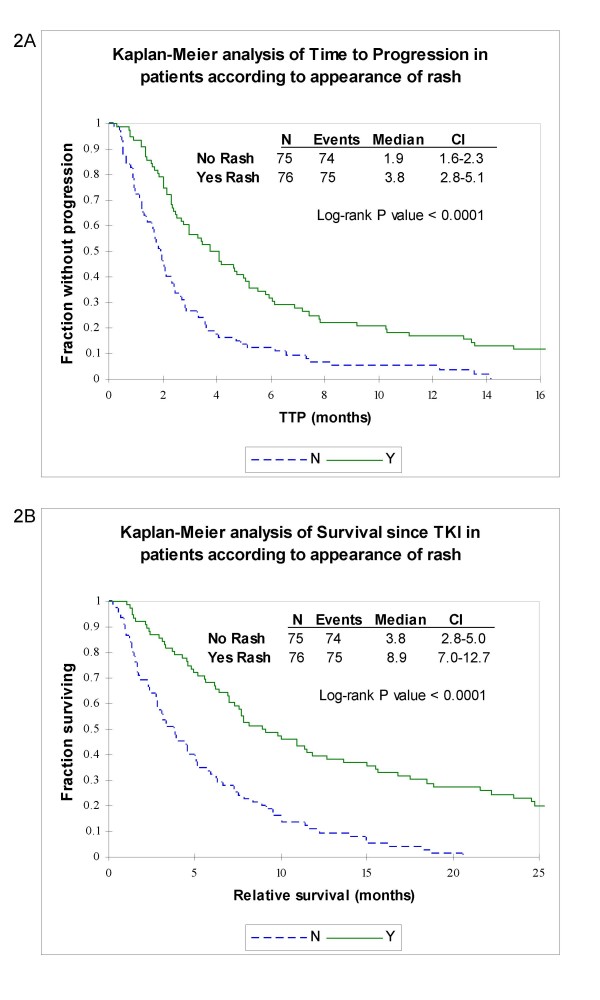
**Rash vs. no rash**. Kaplan-Meier analyses of time to progression (a) and survival since initiating TKI therapy (b) in months among patients that developed a skin rash (solid line) and patients that did not developed rash (dashed line).

Rash was the most common side effect for both groups of patients, and in agreement with previous studies, we found that the development of rash correlated with clinical benefit [16]. The median TTP among patients that experienced a rash was 3.8 months as compared to 1.9 months among the patients that did not experienced a rash. Median survival after the start of therapy was 8.9 months among those with a rash and 3.8 months among those without a rash. Kaplan-Meier analyses indicated that these differences were statistically significant (P value of < 0.0001, Figure 2a-b). Moreover, these differences persisted when the erlotinib and gefitinib data sets were analyzed separately (data not shown).

**Figure 3:**
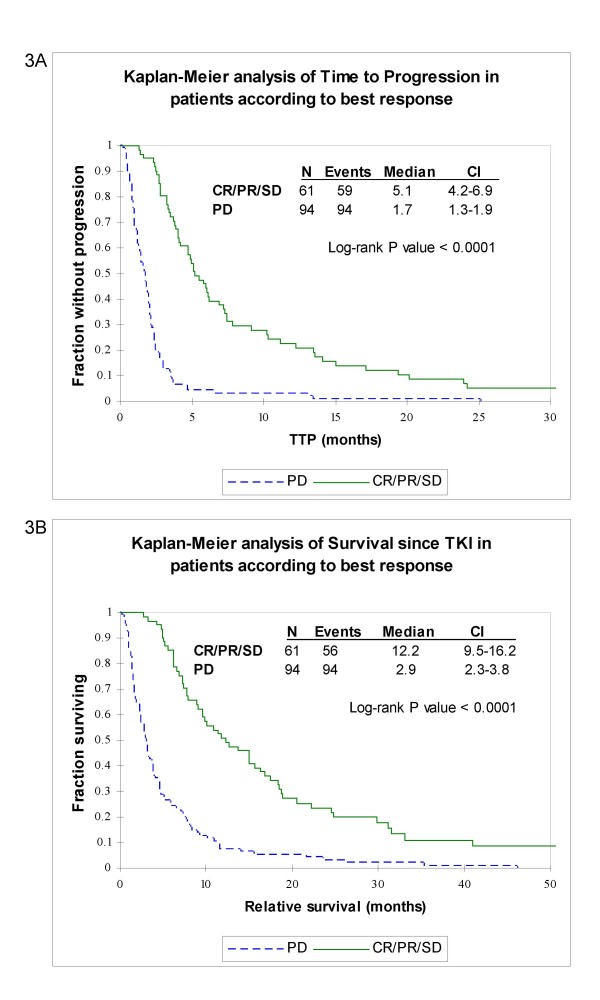
**Disease control vs. progressive disease**. Kaplan-Meier analyses of time to progression (a) and survival since initiating TKI therapy (b) in months among patients that had a response or stable disease (solid line) and patients that had progressive disease (dashed line).

We also investigated outcome in patients that showed progressive disease as compared to those that achieved disease control (defined as CR+PR+SD). Among patients that experienced disease control, the median TTP and the median survival since TKI were 5.1 and 12.2 months, respectively. On the other hand, among the patients that experienced progressive disease, the median TTP and survival since TKI were only 1.7 and 2.9 months, respectively. Kaplan-Meier analyses confirmed that these differences were statistically significant (P value of < 0.0001 Figures 3a-b).

### Immunohistochemistry

**Figure 4:**
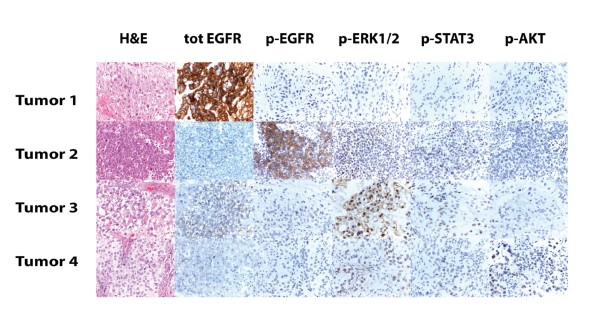
**Immunohistochemistry**. Immunohistochemical analyses of the five protein targets in four representative tumor samples. EGFR (total and phosphorylated) localized to the membrane. pStat 3 localized to nuclei and pErk1/2 and pAkt localized to both nuclei and cytoplasm.

For the 32 patients that had sufficient tissue available, we used immunohistochemistry (IHC) to measure the levels of phosphorylated EGFR (pEGFR), phosphorylated Akt (pAkt), phosphorylated Stat3 (pStat3) and phosphorylated Erk1/2 (pErk1/2) proteins. We also measured the level of total EGFR expression for comparison (See Additional file 1). There were a wide range of protein expression levels. As expected, both total and phosphorylated EGFR, was found to be membrane-associated. Phosphorylated Stat 3 localized to nuclei and pErk1/2 and pAkt localized to nuclei and cytoplasm. In general, the expression level of the phosphorylated proteins was significantly less than that of total EGFR and the signal from phosphorylated antigens was more prominent in tumor cells located toward the periphery of the tissue. Four tumor samples were selected to represent the range of expression observed (Figure 4). Total EGFR was the antigen with the highest expression levels and it was detected in all tumor samples. The second most widely expressed antigen was pErk1/2 followed by pStat3. pEGFR and pAkt were weakly expressed in most samples.

### Association between phospho-protein level and response to gefitinib

**Figure 5:**
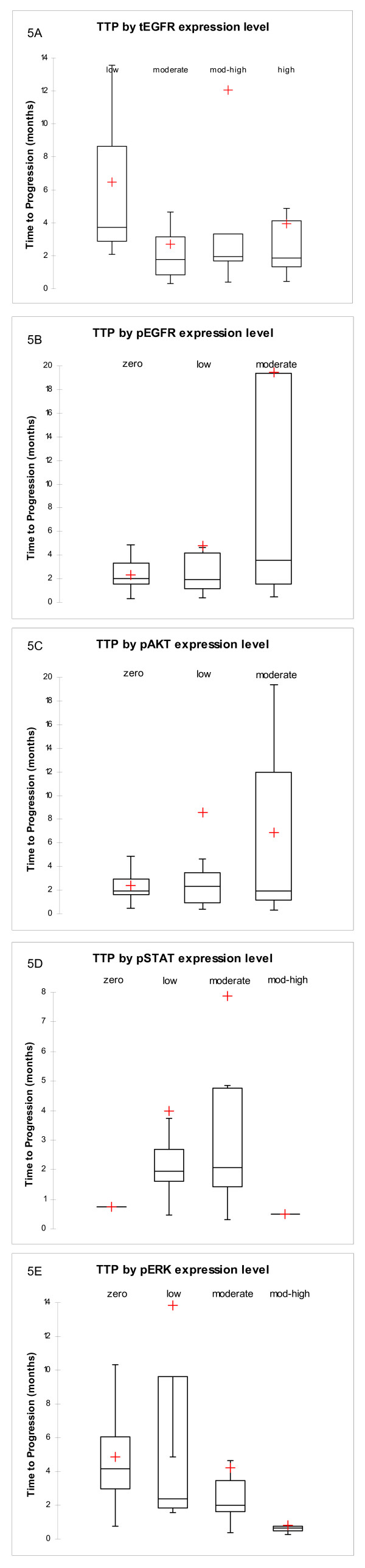
**Time to progression by expression level**. Box Plot Analyses of the association between lengths of time to progression according to the expression levels of a) total EGFR, b) phosphorylated EGFR, c) phosphorylated Akt, d) phosphorylated Stat3 and e) phosphorylated Erk1/2. Time to progression is expressed in months. Expression levels are expressed in four categories zero, low, moderate, moderate-high, and high, where zero represents samples with no detected signal, low for scores between 1 and 10, moderate for scores between 11 and 100, moderate-high for scores between 101 and 200 and high for scores between 201 and 300. Each box represents the TTP values between the 1^st ^and the 3^rd ^quartile. The line across each box represents the median TTP. The dash at the end of the lines represents the maximum and minimum TTP. The crosses represent the mean TTP for each category.

We calculated the mean and median TTP as a function of the expression levels (none, low, moderate, moderate high and high) of our five protein markers, total EGFR, pEGFR, pAkt, pStat3 and pErk1/2. We found no discernable association between TTP and the level of total EGFR. On the other hand, we found a positive association between TTP and the level of pEGFR: in general, the higher the level of this protein, the longer the TTP. Patients with no discernable pEGFR signal had a median TTP of 2.0 months while patients with moderate pEGFR levels had a mean TTP of 3.5 months (Figures 5a-b). pAkt and, to a lesser extent, pStat3 were also slightly positively associated with longer TTP (Figures 5c-d). In contrast, phosphorylated Erk1/2 displayed a negative association with TTP: the higher the level of this protein, the shorter the TTP. Patients with little or no detectable levels of pErk1/2 had a median TTP of 4.2 months while patients with high levels of pErk1/2 had a mean TTP of 0.6 months (Figure 5e). When the levels of the four phospho-proteins were compared amongst each other, we found a strong positive correlation between the level of pEGFR and the level of pStat3 that reached statistical significance (p < 0.0001).

## Discussion

The identification of patients that are likely to benefit from EGFR TKI therapy is a priority for patients with advanced non-small cell lung cancer. A recent study showed that TKI therapy-induced rash strongly correlated with better disease control and progression free survival, and concluded that the development of rash should be viewed as an indication of likelihood of clinical benefit [16]. In this study, we also found that the appearance of rash correlated with longer TTP and survival with TKI treatment. In addition, we found that patients who achieved some degree of disease control at the first response evaluation had a significantly better outcome than patients who experienced disease progression.

Although early clinical indicators of response are useful, the identification of pre-treatment predictors of benefit would be even more desirable. We hypothesized that tumors that are actively using the EGFR pathway for survival and growth would be most susceptible to TKI therapy-mediated inhibition. We also hypothesized that the activation status of the EGFR pathway could be assessed by evaluating the phosphorylation level of key members of the pathway, rather than total protein levels. Accordingly, we determined the expression levels of the activated receptor (pEGFR), and the activated cytosolic end points to the three branches of the EGFR pathway (pAkt, pStat3 and pErk1/2) in pre-treatment samples of tumor tissue from patients undergoing TKI therapy and response assessment.

In general, the expression level of the phosphorylated proteins was significantly less than that of total EGFR. This reflects the fact that the phospho-proteins are more labile and more difficult to detect than total protein levels [17]. In agreement with this, we found that the signal from phosphorylated antigens was more prominent in tumor cells located toward the periphery of the tissue blocks as opposed to cells in more internal regions. We presume that the time delay that occurs from the immersion of the block in fixative to its complete permeation results in the observed pattern of expression. Interestingly, when we compared the levels of the four phospho-proteins amongst each other, we found a significant correlation between the level of pEGFR and pStat3, in general agreement with the notion that the activation of the receptor gets transduced most directly to pStat3 in the signaling pathway.

Analysis of the association between outcome and phospho-protein expression suggested that the pattern of expression may be useful as a predictor of clinical benefit from TKI therapy. However, the small number of samples precluded obtaining statistically significant results. Higher levels of pEGFR, pAkt, and pStat3 were associated with longer TTP, while higher levels of pErk1/2 were associated with shorter TTP. These results make biologic sense given the presumed position of these proteins in the pathway. pAkt and pStat are thought to mediate cell survival, and it is possible that detecting these proteins indicates that a tumor is using the pathway for survival and that inhibition of the pathway provokes widespread apoptosis. On the other hand, pErk, mediates cell proliferation and is downstream of *ras*, a gene that is often mutated in these tumors. High levels of pErk may reflect activation of *ras *rather than EGFR, a condition where EGFR TKI would be ineffective.

## Conclusion

In our experience, erlotinib was found slightly superior to gefitinib in objective response rate and disease control rate, but in terms of time to progression and survival after treatment, the two agents were statistically comparable.

In the absence of molecular predictors of response, the appearance of a rash and evidence of disease control at first follow-up exams are good indicators of response to TKIs. In combination, pEGFR, pAKT, pStat3 and pErk1/2 are promising predictive markers of response to EGFR TKI and deserve further analysis.

## Competing interests

The authors declare that they have no competing interests.

## Authors' contributions

IFE designed and coordinated the study, participated in the immunoassays and their interpretation, participated in the clinical data compilation, participated in the statistical analysis and drafted the manuscript. CB participated in the design of the study, coordinated the clinical data compilation, and participated in the immunoassays and statistical analysis. PA participated in the interpretation of the immunoassays. KC conducted the immunoassays. DH participated in the design of the study and helped draft the manuscript. All authors read and approved the final manuscript.

## Pre-publication history

The pre-publication history for this paper can be accessed here:

http://www.biomedcentral.com/1471-2407/9/333/prepub

## Supplementary Material

Additional file 1 **Immunohistochemistry scores**. Thirty two patients had tissue available for immunohistochemical analysis. Detailed information about each tissue sample is listed on the second column. Patient information, such as performance status (ECOG) and the number of previous lines of chemotherapy at the time of TKI administration is also listed. IHCs were scored on a semiquantitative basis of percentage of positive tumour cells (0-100%), multiplied by staining intensity (0 = negative, 1 = weak, 2 = moderate, 3 = strong). The percentage of cells staining is shown for each antigen. Patients are listed according to increasing length of time to progression. Click here for file
