# Supplementary material for: Response to gefitinib and erlotinib in Non-small cell lung cancer: a retrospective study
Source: BMC Cancer. 2009 Sep 18;9:333. doi: 10.1186/1471-2407-9-333 (PMC2758901; doi:10.1186/1471-2407-9-333)
Supplement: Additional file 1 — Immunohistochemistry scores. Thirty two patients had tissue available for immunohistochemical analysis. Detailed information about each tissue sample is listed on the second column. Patient information, such as performance status (ECOG) and the number of previous lines of chemotherapy at the time of TKI administration is also listed. IHCs were scored on a semiquantitative basis of percentage of positive tumour cells (0-100%), multiplied by staining intensity (0 = negative, 1 = weak, 2 = moderate, 3 = strong). The percentage of cells staining is shown for each antigen. Patients are listed according to increasing length of time to progression. [file 1471-2407-9-333-S1.DOC]

**Additional file 1: Immunohistochemistry scores**

| **Pt** | **Tissue** | **TKI** | **Previous chemo lines** | **ECOG at time of TKI** | **TTP** | **tEGFR score** | **%** | **pEGFR score** | **%** | **pERK1/2 score** | **%** | **pSTAT3 score** | **%** | **pAKT score** | **%** |
| --- | --- | --- | --- | --- | --- | --- | --- | --- | --- | --- | --- | --- | --- | --- | --- |
| 6 | chemo naive IB lung lesion | Gefitinib | 0 | 1 | 9 | 63 | 43 | 0 | 0 | 175 | 80 | 28 | 23 | 15 | 10 |
| 13 | chemo naive IIA lung lesion | Gefitinib | 0 | 2 | 12 | 113 | 60 | 4 | 3 | 19 | 14 | 13 | 12 | 1 | 1 |
| 29 | chemo naive IV lymph node | Gefitinib | 2 | 1 | 14 | 210 | 93 | 0 | 0 | 102 | 76 | 3 | 3 | 0 | 0 |
| 30 | chemo naive IV lymph node | Gefitinib | 1 | 1 | 15 | 195 | 95 | 45 | 45 | 168 | 93 | 120 | 90 | 3 | 3 |
| 3 | chemo naive IB lung lesion | Gefitinib | 0 | 2 | 23 | 75 | 48 | 0 | 0 | 0 | 0 | 0 | 0 | 8 | 8 |
| 25 | chemo naive III lung lesion | Gefitinib | 0 | 2 | 23 | 25 | 23 | 10 | 10 | 130 | 60 | 63 | 45 | 15 | 10 |
| 16 | chemo naive IV lymph node | Gefitinib | 3 | 1 | 24 | 210 | 98 | 10 | 10 | 130 | 75 | 46 | 33 | 1 | 1 |
| 26 | IIIA lung lesion post 4 cycles neoadj chemo | Gefitinib | 1 | 0 | 36 | 55 | 60 | 7 | 7 | 20 | 10 | 5 | 5 | 0 | 0 |
| 22 | chemo naive IIIA lymph node | Gefitinib | 2 | 2 | 38 | 300 | 100 | 10 | 10 | 20 | 20 | 68 | 30 | 5 | 5 |
| 10 | chemo naive IV brain met | Gefitinib | 1 | 2 | 48 | 275 | 100 | 20 | 10 | 29 | 13 | 4 | 3 | 3 | 3 |
| 31 | chemo naive IIB lung lesion | Erlotinib | 1 | 1 | 48 | 215 | 95 | 0 | 0 | 1 | 1 | 13 | 13 | 0 | 0 |
| 12 | chemo naive IIB lung lesion | Gefitinib | 0 | 2 | 50 | 30 | 13 | 1 | 1 | 2 | 1 | 2 | 1 | 20 | 6 |
| 15 | chemo naive IIIB lymph node | Gefitinib | 2 | 1 | 52 | 110 | 68 | 0 | 0 | 17 | 17 | 1 | 1 | 0 | 0 |
| 17 | chemo naive IA lung lesion | Gefitinib | 0 | 0 | 58 | 103 | 63 | 10 | 10 | 72 | 50 | 80 | 65 | 48 | 26 |
| 21 | chemo naive IV lymph node | Gefitinib | 2 | 0 | 59 | 55 | 50 | 1 | 1 | 5 | 5 | 10 | 10 | 0 | 0 |
| 1 | chemo naive IV bone met | Gefitinib | 2 | 0 | 60 | 208 | 98 | 0 | 0 | 11 | 5 | 14 | 8 | 0 | 0 |
| 7 | chemo naive IB lung lesion | Gefitinib | 1 | 2 | 62 | 80 | 45 | 0 | 0 | 12 | 5 | 15 | 9 | 3 | 3 |
| 2 | chemo naive IB lung lesion | Gefitinib | 1 | 1 | 63 | 20 | 10 | 0 | 0 | 2 | 1 | 14 | 11 | 0 | 0 |
| 5 | chemo naive IIIA lung lesion | Gefitinib | 4 | 2 | 65 | 250 | 100 | 1 | 1 | 158 | 80 | 6 | 6 | 0 | 5 |
| 8 | chemo naive IB lung lesion | Gefitinib | 3 | 1 | 81 | 280 | 100 | 1 | 1 | 27 | 15 | 4 | 3 | 5 | 5 |
| 14 | IIIA lung lesion post 3 cycles neoadj chemo | Gefitinib | 4 | 1 | 82 | 175 | 83 | 0 | 0 | 1 | 1 | 6 | 6 | 1 | 1 |
| 28 | chemo naive IV lung lesion | Gefitinib | 2 | 0 | 101 | 133 | 65 | 0 | 0 | 78 | 38 | 12 | 7 | 5 | 5 |
| 19 | chemo naive IB lung lesion | Gefitinib | 0 | 1 | 108 | 76 | 51 | 11 | 8 | 44 | 19 | 14 | 10 | 3 | 3 |
| 11 | chemo naive IIB lung lesion | Gefitinib | 0 | 2 | 114 | 3 | 3 | 0 | 0 | 0 | 0 | 1 | 1 | 0 | 0 |
| 4 | chemo naive IB lung lesion | Gefitinib | 0 | 1 | 141 | 260 | 100 | 0 | 0 | 0 | 0 | 16 | 11 | 0 | 0 |
| 18 | chemo naive IIIB lung lesion | Gefitinib | 0 | 1 | 142 | 55 | 25 | 2 | 1 | 70 | 35 | 42 | 20 | 3 | 4 |
| 20 | chemo naive IV lung lesion | Gefitinib | 0 | 0 | 148 | 265 | 100 | 0 | 0 | 1 | 1 | 32 | 21 | 0 | 0 |
| 27 | chemo naive IV lung lesion | Gefitinib | 1 | 2 | 315 | 53 | 50 | 2 | 3 | 0 | 0 | 34 | 37 | 11 | 8 |
| 24 | chemo naive IIIB lung lesion | Gefitinib | 0 | 0 | 414 | 3 | 3 | 2 | 2 | 13 | 7 | 19 | 10 | 15 | 8 |
| 32 | chemo naive IB lung lesion | Erlotinib | 0 | 1 | 588 | 192 | 87 | 39 | 27 | 15 | 10 | 38 | 18 | 19 | 10 |
| 23 | chemo naive IIIA lung lesion | Gefitinib | 2 | 1 | 731 | 193 | 95 | 1 | 1 | 9 | 5 | 1 | 1 | 10 | 7 |
| 9 | chemo naive IV lung lesion | Gefitinib | 2 | 1 | 2081 | 139 | 93 | 9 | 7 | 10 | 10 | 32 | 24 | 3 | 2 |

Additional file 1. Thirty two patients had tissue available for immunohistochemical analysis. Detailed information about each tissue sample is listed on the second column. Patient information, such as performance status (ECOG) and the number of previous lines of chemotherapy at the time of TKI administration is also listed. IHCs were scored on a semiquantitative basis of percentage of positive tumour cells (0-100%), multiplied by staining intensity (0=negative, 1= weak, 2=moderate, 3=strong). The percentage of cells staining is shown for each antigen. Patients are listed according to increasing length of time to progression.
